# GGT1 is a SNP eQTL gene involved in STAT3 activation and associated with the development of Post-ERCP pancreatitis

**DOI:** 10.1038/s41598-024-60312-2

**Published:** 2024-05-28

**Authors:** Ryutaro Furukawa, Masaki Kuwatani, Jing-Jing Jiang, Yuki Tanaka, Rie Hasebe, Kaoru Murakami, Kumiko Tanaka, Noriyuki Hirata, Izuru Ohki, Ikuko Takahashi, Takeshi Yamasaki, Yuta Shinohara, Shunichiro Nozawa, Shintaro Hojyo, Shimpei I. Kubota, Shigeru Hashimoto, Satoshi Hirano, Naoya Sakamoto, Masaaki Murakami

**Affiliations:** 1 Division of Molecular Psychoneuroimmunology, Institute for Genetic Medicine and Graduate School of Medicine, Hokkaido University, Kita-15, Nishi-7, Kita-Ku, Sapporo, 060-0815 Japan; 2 Department of Gastroenterology and Hepatology, Hokkaido University Faculty of Medicine and Graduate School of Medicine, Sapporo, Japan; 3 Institute of Preventive Genomic Medicine, School of Life Sciences, Northwest University, Xian, China; 4 Quantum Immunology Team, Institute for Quantum Life Science, National Institutes for Quantum Science and Technology (QST), Chiba, Japan; 5 Division of Molecular Neuroimmunology, National Institute for Physiological Sciences, National Institutes of Natural Sciences, Okazaki, Japan; 6 Department of Gastroenterological Surgery II, Hokkaido University Faculty of Medicine, Sapporo, Japan; 7 Institute for Vaccine Research and Development (HU-IVReD), Hokkaido University, Sapporo, Japan

**Keywords:** Immunology, Cytokines, Inflammation

## Abstract

Post-ERCP pancreatitis (PEP) is an acute pancreatitis caused by endoscopic-retrograde-cholangiopancreatography (ERCP). About 10% of patients develop PEP after ERCP. Here we show that gamma-glutamyltransferase 1 (GGT1)-SNP rs5751901 is an eQTL in pancreatic cells associated with PEP and a positive regulator of the IL-6 amplifier. More PEP patients had the GGT1 SNP rs5751901 risk allele (C) than that of non-PEP patients at Hokkaido University Hospital. Additionally, GGT1 expression and IL-6 amplifier activation were increased in PEP pancreas samples with the risk allele. A mechanistic analysis showed that IL-6-mediated STAT3 nuclear translocation and STAT3 phosphorylation were suppressed in GGT1-deficient cells. Furthermore, GGT1 directly associated with gp130, the signal-transducer of IL-6. Importantly, GGT1-deficiency suppressed inflammation development in a STAT3/NF-κB-dependent disease model. Thus, the risk allele of GGT1-SNP rs5751901 is involved in the pathogenesis of PEP via IL-6 amplifier activation. Therefore, the GGT1-STAT3 axis in pancreas may be a prognosis marker and therapeutic target for PEP.

## Introduction

Acute pancreatitis is a serious disorder, leading to hospitalizations and deaths. Moreover, incidences have more than doubled in the past 30 years^1^. Post-ERCP pancreatitis (PEP) is an acute pancreatitis that occurs in about 10% of patients who undergo endoscopic retrograde cholangiopancreatography (ERCP) and is fatal in about 1% cases^2^. Although non-steroidal anti-inflammatory drugs and pancreatic duct stenting have been shown to reduce PEP, there remains a need to understand the molecular mechanisms and to find additional and more effective methods for PEP prevention.

Although no reports have shown the genetic predisposition of PEP, genome wide association study (GWAS) showed thirteen genes (SPINK1, CTRC, CASR, PRSS1, CLDN2, CPA1, GGT1, CEL, FUT2, ABO, CFTR, MORC4, CTRB1-2) are related to chronic pancreatitis^3–13^, and six of them (SPINK1, CTRC, PRSS1, CLDN2, MORC4, GGT1) were also associated with acute pancreatitis^14–18^.

Glutamyltransferase 1 (GGT1) is abundantly expressed in various tissues including the pancreas. The main function of normal GGT1 is to induce glutathione molecules followed by providing protection against oxidative stress^19^. It is known that oxidative stress plays a role in the pathogenesis of acute pancreatitis^20^. Additionally, smokers with the GGT1 SNP rs5751901 show a higher risk for acute pancreatitis^17^. Furthermore, GGT1 is involved in NF-κB pathway in a manner dependent on reactive oxygen species (ROS) regulation^21^, although GGT1-mediated glutathione molecules suppressed oxidative stress^19^. Therefore, it is not known whether GGT1 directly plays a role for NF-κB activation.

We have reported that the IL-6 amplifier is a molecular mechanism for inflammation induction in various inflammatory diseases^22,23^. The IL-6 amplifier, which is an enhanced NF-κB activation in nonimmune cells, is induced by the simultaneous activation of two transcriptional factors, STAT3 and NF-κB, in nonimmune cells including ubiquitously expressed cells including fibroblasts and endothelial cells and tissue-specific cells such as kidney tubular cells, keratinocytes, and chondrocytes^24–28^.

Moreover, these results suggested that a main STAT3 stimulator is IL-6, while there are several NF-κB stimulating factors involved in each disease, and these factors can change with the phase of the disease. The IL-6 amplifier leads to the excessive expression of NF-κB targets, including inflammatory cytokines, chemokines, and growth factors, followed by the disruption of homeostasis via immune cell infiltration and nonimmune cell proliferation in the lesions to cause chronic inflammation. A genome-wide shRNA screening identified GGT1 as one of more than 1000 genes that positively regulate the IL-6 amplifier^29^. In this study, we investigated the prognosis and therapeutic potential of GGT1 in PEP.

## Results

### Patient background

A total of 24 patients in the PEP group and 85 patients in the non-PEP group were enrolled in this study in Hokkaido University Hospital. In addition to age, sex, and primary disease, 11 of the 12 identified risk factors for PEP were compared between the two groups, excluding “female”. The results are shown in Table 1. Only "repetitive pancreatic guidewire cannulation" was significantly more common in the PEP group (*p* = 0.02), while "pancreatic injection" and "EPLBD" tended to be more common (*p* = 0.07 and *p* = 0.06, respectively).

**Table 1:** Patient background (before propensity score matching).

	PEP (n = 24)	Non-PEP (n = 85)	*P* value
Age Median (range)	69.5 (44–92)	70 (27–90)	0.59
M/F	12/12	50/35	0.44
Disease (PC/Other cancer/benign)	8/10/6	21/38/26	0.68
Prior PEP	0	1	0.63
Previous recurrent pancreatitis	0	2	0.45
Suspected SOD	0	0	–
Younger patient age (< 40)	0	2	0.45
Absence of chronic pancreatitis	24	85	–
Normal serum bilirubin	10	38	0.79
Difficult cannulation	12	37	0.57
Repetitive pancreatic guidewire cannulation	16	34	0.02
Pancreatic injection	8	14	0.07
Pancreatic sphincterotomy	2	4	0.49
EPLBD	2	1	0.06

Propensity score matching was performed using EZR^30^. The objective variable was the presence of PEP, and the explanatory variables were age, gender, and 11 PEP risk factors (excluding “female”). Twenty cases in each group were matched (Table 2). No significant differences were found in the groups (Table 2).

**Table 2:** Patient background (after propensity score matching).

	PEP (n = 20)	Non-PEP (n = 20)	*P* value
Age Median (range)	70 (41–84)	70 (27–90)	0.95
M/F	10/10	5/15	0.10
Disease (PC/Other cancer/benign)	8/10/6	21/38/26	0.72
Prior PEP	0	0	–
Previous recurrent pancreatitis	0	0	–
Suspected SOD	0	0	–
Younger patient age (< 40)	0	0	–
Absence of chronic pancreatitis	20	20	–
Normal serum bilirubin	10	6	0.20
Difficult cannulation	11	11	1
Repetitive pancreatic guidewire cannulation	14	13	0.74
Pancreatic injection	8	6	0.52
Pancreatic sphincterotomy	1	3	0.29
EPLBD	2	0	0.15

PC: pancreatic cancer, SOD: sphincter of Oddi dysfunction, EPLBD: endoscopic papillary large balloon dilation.

### GGT1 SNP rs5751901 was associated with PEP

**Figure 1:**
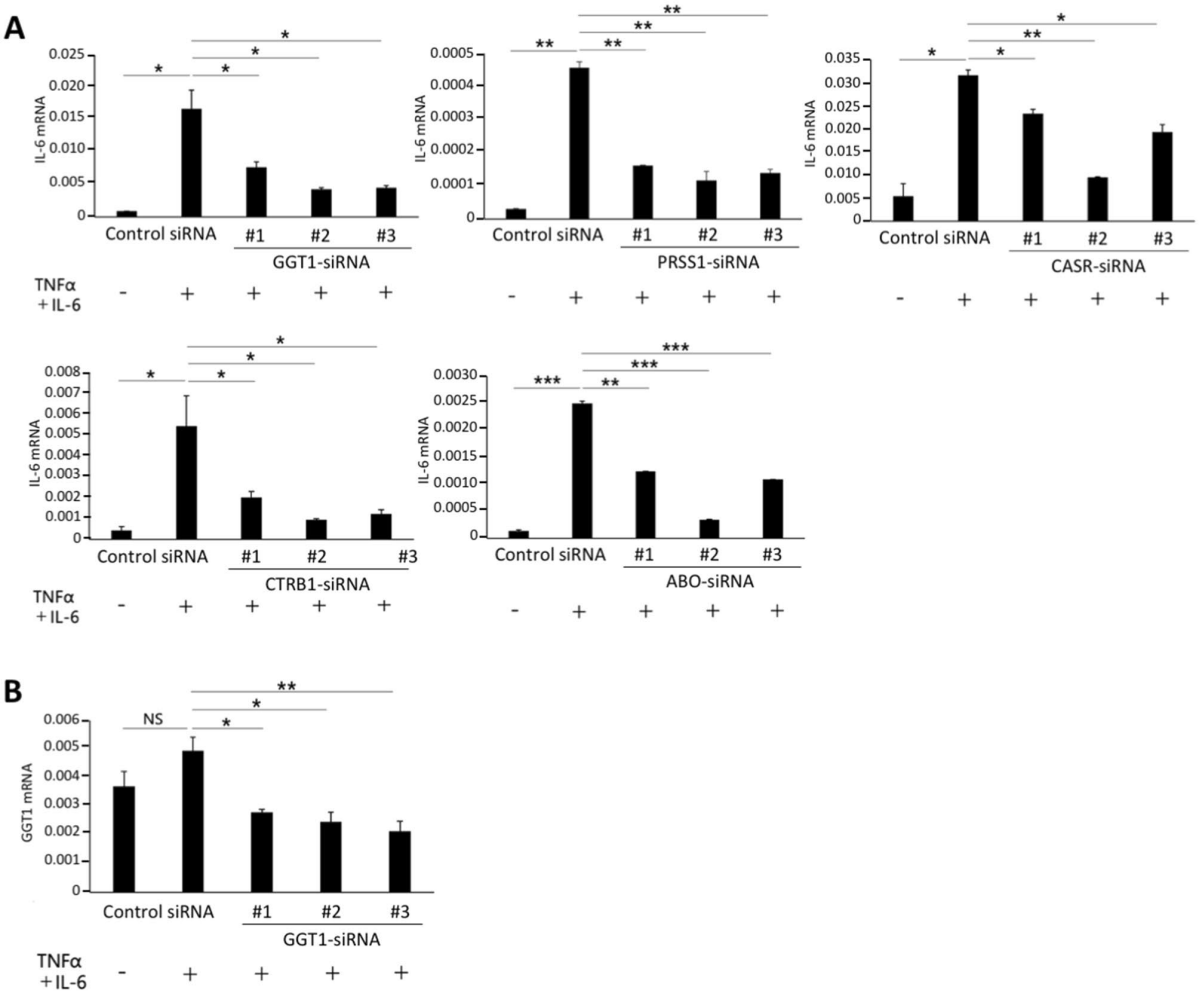
Five pancreatitis-related genes are positive-regulators for the IL-6 amplifier. (**A**) H4 cells were transfected with three siRNAs for each gene. qPCR was performed after stimulation with TNF-α plus IL-6. The results of five genes whose siRNA suppressed IL-6 are shown. (**B**) GGT1 mRNA expression after transfecting H4 cells with three different GGT1-siRNAs and stimulating them with TNF-α plus IL-6. Means ± SEM are shown. *p < 0.05, **p < 0.01, ***p < 0.005; NS, not significant (Student’s t-test).

Thirteen genes (GGT1, PRSS1, CASR, CTRB1, ABO, CTRC, CLDN2, CPA1, FUT2, MORC4, SPINK1, CEL, or CFTR) have been previously identified as chronic/acute pancreatitis-related genes^3–18^, and 9 of them (GGT1, CTRB1, ABO, CTRC, CLDN2, CPA1, FUT2, CEL, CFTR) are potential regulators of the IL-6 amplifier by our results, which was reported previously^29^. The expression of IL-6 mRNA was investigated in H4 cells (human neuroglioma cells) treated with three siRNAs for each gene in the presence or absence of cytokine stimulation (TNF-α and IL-6). Cytokine-induced IL-6 mRNA expression was significantly suppressed in cells with GGT1, PRSS1, CASR, CTRB1, or ABO siRNA (Fig. 1A) but not with CTRC, CLDN2, CPA1, FUT2, or MORC4 siRNA (Supplementary Fig. 1A). The IL-6 mRNA was partially suppressed in cells with SPINK1, CEL, or CFTR siRNA (Supplementary Fig. 1B). Additionally, the expression of GGT1 was suppressed by siRNA (Fig. 1B). Therefore, GGT1, PRSS1, CASR, CTRB1, and ABO are positive regulators of the IL-6 amplifier.

**Figure 2:**
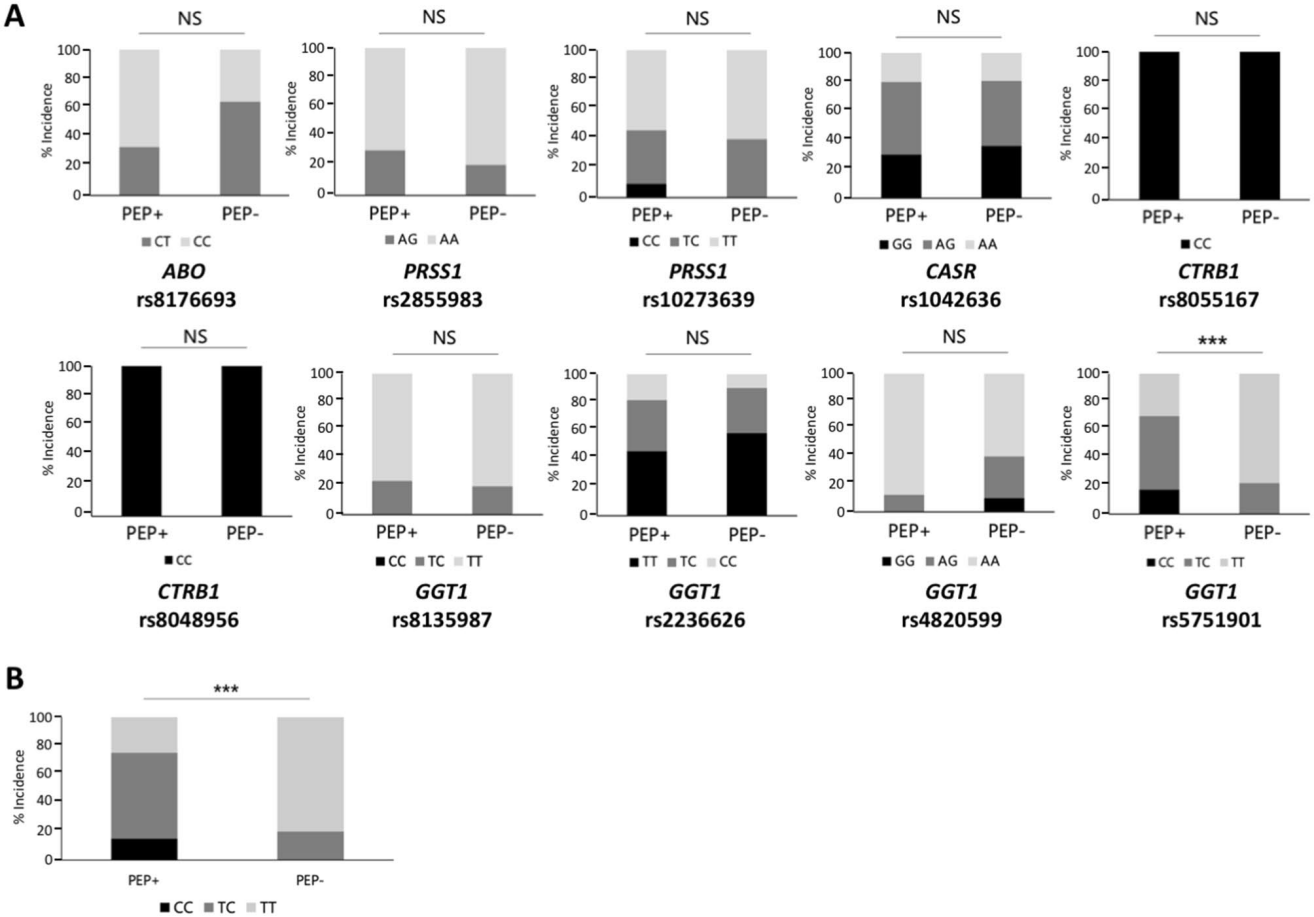
GGT1 SNP rs5751901 is associated with PEP. Possession rate of each SNP in the subjects. (**A**) DNA was extracted from blood, DNA sequencing was performed for each SNP, and possession rates were compared between the two groups. (**B**) We examined the prevalence of GGT1 SNP rs5751901 in a group adjusted for patient background by propensity score matching by risk factors for pancreatitis. For each disease-associated gene, risk SNPs were indicated by the degree of color. Black indicated high-risk SNP homozygotes, dark gray indicated high-risk SNP heterozygotes, and light gray indicated no high-risk SNP. Means ± SEM are shown. ***p < 0.005 (Student’s t-test).

Because we showed that some disease-associated genes for Sjogren’s disease and keloids are involved in activation of the IL-6 amplifier^24,27^, we hypothesized that SNPs of the above genes are associated with PEP development in our cohort. There were ten SNPs, including rs8176693 (ABO), rs2855983 and rs10273639 (PRSS1), rs1042636 (CASR), rs8055167 and rs8048956 (CTRB1), rs8135987, rs2236626, rs4820599, and rs5751901 (GGT1). Among them, we found that risk alleles (C/C and T/C) of GGT1 SNP rs5751901 are increased in PEP patients compared with non-PEP patients (16/24 cases in the PEP group, 19/85 cases in the non-PEP group, *p* < 0.001) (Fig. 2A, risk SNPs were indicated by the degree of color. Black indicated high-risk SNP homozygotes, dark gray indicated high-risk SNP heterozygotes, and light gray indicated no high-risk SNP**)**. Of the 20 patients in the PEP group and 20 patients in the non-PEP group extracted by propensity score matching, the percentage with risk allele C was significantly higher in the PEP group than in the non-PEP group (15/20 in the PEP group and 4/20 in the non-PEP group, *p* < 0.001) (Fig. 2B). Thus, GGT1 SNP rs5751901 is associated with PEP development in our cohort.

### The risk allele of GGT1 SNP rs5751901 increased the expression of GGT1 and enhanced activation of the IL-6 amplifier in the pancreas

**Figure 3:**
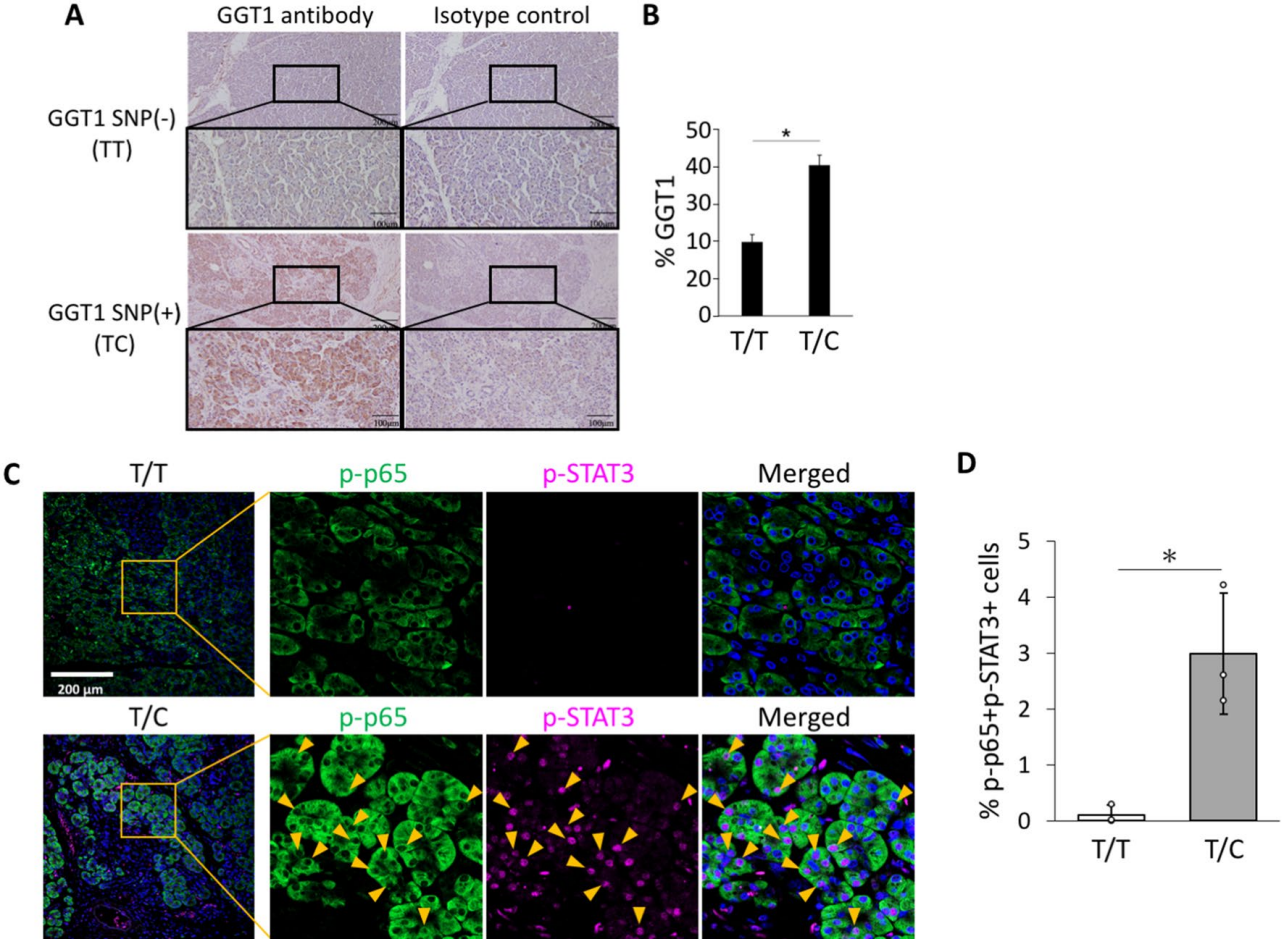
The risk allele of GGT1 SNP rs5751901 increases the expression of GGT1 molecules and enhances activation of the IL-6 amplifier in the pancreas. (**A**) Pancreatic tissues from patients with (T/T, n = 5) and without (T/C, n = 3) the risk allele were immunohistochemically stained using anti-GGT1 antibody. (**B**) The percentage of GGT1 positive cells. More than 200 cells were measured in each section at three or more arbitrary sites. (**C**) Pancreatic tissues from patients with (T/T, n = 5) and without (T/C, n = 3) the risk allele were immunohistochemically stained using anti-p-p65 and -p-STAT3 antibodies. (**D**) The percentage of double-positive cells. More than 200 cells were measured in each section at three or more arbitrary sites. Means ± SEM are shown. *p < 0.05 (Student’s t-test).

We considered two explanations for how the risk alleles of GGT1 rs5751901 SNP enhance activation of the IL-6 amplifier in pancreatic cells: (i) alternative splicing and (ii) eQTL. Previously, we reported that a NEDD4 SNP induces a specific alternative splice in keloid patients^24^ and a GTF2i SNP acts as an eQTL in patients with Sjogren’s disease^27^. There are 26 splicing variants in GGT1 mRNA, but we investigated the expression levels of only the coding exons. No obvious differences in the expression patterns of each exon was found between SNP-(T/T), SNP + (T/C) and SNP + (C/C) groups (data not shown). On the other hand, according to the GTEx portal site (https://gtexportal.org/home/), the expression of GGT1 rs5751901 SNP increases in the order of SNP-(T/T), SNP + (T/C) and SNP + (C/C), suggesting an eQTL. We then performed immunohistochemistry experiments using anti-GGT1 antibody and pancreas samples with or without the risk SNP. We found that GGT1 positive cells are increased in the pancreas with the risk SNP relative to those without (Fig. 3A, B). These results show that the risk allele of GGT1 SNP rs5751901 increases the expression of GGT1 molecules in the pancreatic cells.

We then performed immunofluorescence staining in sections of the pancreas with or without the risk GGT1 rs5751901 SNP using anti-phosphorylated p65 (p-p65) and anti-phosphorylated STAT3 (p-STAT3) antibodies to investigate the activation of the IL-6 amplifier in pancreas. We found that p-p65 and p-STAT3 double positive pancreatic cells are significantly increased in PEP patients with the risk alleles (Fig. 3C, D), suggesting that activation of the IL-6 amplifier is enhanced in a manner dependent on the risk SNP in the pancreas. Thus, the risk alleles of GGT1 rs5751901 SNP were significantly associated with activation of the IL-6 amplifier in the pancreas.

**Figure 4:**
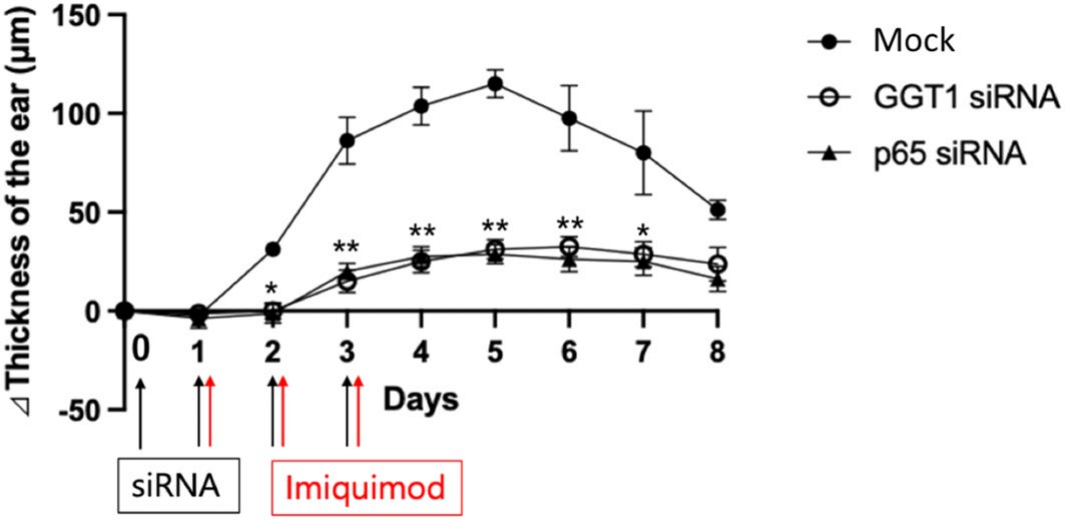
GGT1 is involved in the development of NF-κB-induced inflammation in vivo. Ear thickening in mice caused by psoriasis-like dermatitis following imiquimod treatment. One ear of each mouse was used, and two mice were used for each siRNA (n = 4). siRNA (mock, GGT1, p65) was applied to the auricle on days 0–3, and imiquimod was applied on days 1–3. The thickness of the auricle was measured. Means ± SEM are shown. *p < 0.05, **p < 0.01.

We then investigated that GGT1 increase is indeed involved in the inflammation development in vivo. We employed a NF-κB-mediated inflammation model using a TLR7 ligand, imiquimod, and investigated GGT1 function in vivo. Local GGT1 siRNA and p65 siRNA treatment significantly suppressed the development of the model (Fig. 4), demonstrating GGT1’s involvement in the development of NF-κB-induced inflammation in vivo.

## Discussion

We here investigated a molecular mechanism of PEP, which is one of acute pancreatitis after ERCP. Over ten pancreatitis-associated genes had been reported recently, while there is no research which shows whether they are involved in the development of PEP. We have been studying the IL-6 amplifier, which is a fundamental molecular machinery of inflammation in nonimmune cells and here investigated the relationship between PEP and the IL-6 amplifier activation by using pancreatitis-associated genes. We firstly suppressed the expression of these pancreatitis-associated genes using siRNA in nonimmune cells and examined the expression of IL-6 mRNA to identify whether these genes are regulators of the IL-6 amplifier, and found that GGT1 is a possible positive regulator of the IL-6 amplifier at least in vitro. We then investigated the SNPs of several candidate genes in a cohort at Hokkaido University, and found that GGT1 SNP rs5751901 is significantly associated with PEP development. Consistently, the risk alleles of GGT1 SNP rs5751901 were associated with patients having PEP and increased GGT1 expression in the pancreas. Moreover, we showed that GGT1 regulated the activation of the IL-6 amplifier in PEP pancreas. Furthermore, GGT1 deficiency suppressed a NF-κB-mediated in vivo inflammatory disease model. These results suggest that the risk alleles of GGT1 SNP rs5751901 are an eQTL in pancreatic cells and a positive regulator of the IL-6 amplifier, followed by regulating the development of PEP.

The main function of GGT1 is to induce glutathione molecules via cysteine synthesis followed by indirectly associated with inflammation^19^. However, GGT1 is sometimes associated with inflammation responses. For example, GGT1 catalyzes the conversion of leukotriene (LT), an inflammatory lipid mediator, from LTC4 to LTD4. This catalysis leads to inflammation responses including enhancement of endothelial cell adhesion and chemokine production in mast cells^31^. Additionally, the GGT1-dependent production of reactive oxygen species induces the activation of NF-κB^21^. We here showed that GGT1 is involved in PEP development via the IL-6 amplifier, NF-κB activator in pancreatic cells. These findings suggest that GGT1-NF-κB axis is both a prognosis and therapeutic candidate for PEP.

Recently Corti et al.^32^ reported that the differential S-nitrosoglutathione (GSNO) catabolism mediated by GGT enzyme activity can downregulate the production of IL-8, which is a NF-κB target. Consistently, IL-8 levels were associated with pancreatitis risk^33^*.* Therefore, the role played by GGT1 in the metabolism of endogenous GSNO may also deserve attention insofar as the possibility exists that selected polymorphisms of the GGT1 enzyme are associated with lower activation of this endogenous anti-inflammatory molecule, as suggested previously^34^.

The pancreas has various cell populations including ductal cells, exocrine glandular cells, fibroblasts, macrophages, pancreatic endocrine cells, and endothelial cells. We did an analysis using single-cell analysis data from the human protein atlas (https://www.proteinatlas.org/) showed that pancreas cells are divided into 17 populations, although there are some mixed cell types. We investigated the expression of pancreatitis-associated genes with the ability to act as positive regulators of the IL-6 amplifier; these genes include GGT1, PRSS1, CASR, CTRB1, ABO, SPINK1, and CTRC (Fig. 1A and Supplementary Fig. 1). Interestingly, duct cells expressed mainly GGT1, CASR, and ABO; exocrine glandular cells expressed almost all genes including GGT1, PRSS1, CTRB1, ABO, SPINK1, and CTRC; pancreatic endocrine cells expressed GGT1, CASR, ABO, and SPINK1; fibroblasts expressed GGT1 and ABO; and endothelial cells expressed ABO. Because GGT1 expression was broad in pancreatic cells and ERCP, which inserts an endoscope into the bile duct, sometimes physically damages the duct cells to activate NF-κB, it is reasonable that GGT1 is also associated with triggers in duct cells and the exacerbation in other cells of PEP pathogenesis (Supplementary Fig. 2).

We did not show how GGT1 activates NF-κB pathway. There are several possibilities: (i) GGT1 directly associated with signaling molecules involved in NF-κB and/or STAT3 pathway and (ii) alteration of antioxidant capacity, because GGT1 expression is part of a cell-wide insult initiated and transcription factor driven transformation of cells as part of a stress response, in which many factors are linked to this general response. It is possible that supplying antioxidants (e.g. NAC) prior to injury in cases alter the environment such that the factors driving PEP are quelled, although further detailed study is needed.

This study has several limitations. The first is the small number of cases. Although we found significant differences in the presence of the GGT1 SNP rs5751901 between groups, other SNPs may also show significant differences if the number of cases is increased. In addition, although the present study was conducted at a single institution and was restricted to PEP, more reliable results will be obtained if cases of alcoholic and gallstone acute pancreatitis as well as PEP samples are collected at multiple institutions. Finally, we did not investigate whether GGT1 SNP rs5751901 is involved in the development of pancreatitis in vivo. Instead, we employed a NF-κB-mediated dermatitis, because we did not have a model of pancreatitis, and we wished to investigate whether GGT1-mediated NF-κB activation plays a role in the development of inflammatory diseases in vivo via the IL-6 amplifier activation.

In summary, we demonstrated that the risk allele of GGT1 SNP rs5751901 is involved in the pathogenesis of PEP by increasing GGT1 expression in the pancreas, which leads to activation of the IL-6 amplifier in the pancreas. Therefore, GGT1 SNP rs5751901 and the GGT1-NF-κB axis may be prognosis markers and therapeutic targets for PEP.

## Materials and methods

### Patients and diagnosis

Informed consent was obtained from all patients for the conduct of this study. And the study was conducted in accordance with the Declaration of Helsinki (1964) and approved by the Medical Ethics Committee of the Hokkaido University School of Medicine (IRB#019–0440). Patients who underwent ERCP at the Department of Gastroenterology, Hokkaido University Hospital, between October 29, 2019, and September 24, 2021, and gave consent to participate in this study were included. Patients who developed pancreatitis after ERCP were defined as the PEP group, and those who did not develop pancreatitis were defined as the non-PEP group. PEP was diagnosed when abdominal pain accompanied by elevated serum amylase or lipase levels (more than three times the upper limit of normal) was observed after ERCP according to criteria defined by the American Society for Gastrointestinal Endoscopy^35^. Human pancreatic tissues are derived from patients who underwent pancreatectomy among those who developed PEP (PEP group) and those who did not develop PEP (non-PEP group). Normal pancreatic portions were obtained from the same samples (i.e., all pancreatic tissue samples were from the research cohort in this paper). We have genotyped these human specimens and pathologically examined the relationship between GGT-related genes, PEP, and IL-6 amplifier. Approximately 10 mL peripheral blood (EDTA preserved whole blood) was collected from each patient and used for DNA and RNA extraction. From patients who underwent pancreatic resection, a portion of the resected fresh pancreatic tissue and block fragments after formalin fixation were used.

### Cell lines

The human glioma cell line H4 was purchased from ATCC (Sumitomo Pharma International). The cells were grown in Dulbecco's Modified Eagle's Medium (DMEM; Life Technologies) with 10% fetal bovine serum (FBS) (Thermo Scientific) and incubated at 37 °C and 5% CO_2_. The instruments used for culture were sterilized by autoclaving, and all operations were performed aseptically in a clean bench.

### Real-time PCR

The expression levels of target mRNA and internal control mRNA (glycerol-3-phosphatase dehydrogenase : GAPDH) were quantified using a 7300 fast real-time PCR system (Applied Biosystems) and SYBR Green PCR master mix (KAPA Biosystems). H4 cells were seeded at 1.0 × 10^4^ cells/well in 96-well plates and transfected with 0.5 μL of siRNA (5 μM) using Lipofectamine RNAiMAX (Thermo Fisher Scientific). After 2 h of serum starvation, 100 ng/mL human IL-6 (Toray), 100 ng/mL soluble IL-6 receptor (IL-6R; PeproTech), and 50 ng/mL human TNF-α (PeproTech) were added to the cells and incubated for 3 h. Total RNA was prepared from the cells using a SuperPrep® Cell Lysis & RT Kit for qPCR (TOYOBO). Then, M-MLV Reverse Transcriptase (Promega) was used to synthesize cDNA according to the standard protocol and used for qPCR. The primer sequences used for qPCR are shown in Table S1.

### Human small interfering RNAs

Small interfering RNAs (siRNAs) were transfected into H4 cells using Lipofectamine RNAiMAX (Thermo Fisher Scientific). A real-time (RT) PCR analysis of the respective target was performed. The sequences for the sense oligonucleotides of the most effective knockdown constructs are as follows: human si-GGT1 (1: CAACAGCACCACACGAAAAtt; 2: CCAAGGAACCUGACAACCAtt; 3: UCAACAUCCUCAAAGGGUAtt; Ambion Silencer Select siRNA, Themo Fisher Scientific), human si-p65 (Ambion Silencer Select RELA siRNA, Themo Fisher Scientific) and human si-non-target (Ambion Negative Control #1 siRNA, Themo Fisher Scientific).

### SNP genotyping

Genomic DNA was extracted using the QIAamp DNA Blood Mini Kit (QIAGEN) according to a standard protocol. The extracted genomic DNA was sequenced using primers (Table S2) designed for SNPs in chronic pancreatitis-related genes that were found to be associated with IL-6 amplification. PCR was performed using KOD Fx (Toyobo Life Science) on 100–200 ng of genomic DNA, and after confirming amplification by electrophoresis, the PCR products were purified using a Wizard® SV Gel and PCR Clean-Up System (Promega). Sequential PCR was performed on the purified PCR products using the BigDye® Terminator v3.1 Cycle Squencing Kit (Applied Biosystems). In Fig. 2, risk SNPs were indicated by the degree of color. Black indicated high-risk SNP homozygotes, dark gray indicated high-risk SNP heterozygotes, and light gray indicated no high-risk SNP.

### Immunohistochemistry

Sections of surgically removed pancreatic tissue were prepared by formaldehyde fixation and cut out at 5 μm thickness from paraffin-embedded blocks. After deparaffinization with xylene, the specimens were completely dehydrated with anhydrous ethanol. After washing with ultrapure water, the specimens were immersed in sodium citrate buffer (pH 6.0) and boiled for 10 min for antigen activation. After 30 min at room temperature, the cells were washed with ultrapure water and treated with 3% hydrogen peroxide for 5 min to inactivate endogenous enzymes. Anti-p-p65 antibody (Sigma-Aldrich), anti-p-STAT3 antibody (Cell Signaling Technology), and anti-GGT1 antibody (Sigma-Aldrich) were used as primary antibodies, and biotinylated anti-rabbit IgG monoclonal antibody was used as a secondary antibody. DAB (ImmPACTTM DAB Peroxidase Substrate Kit, Vector Laboratories) was used as the chromogenic substrate.

### Observation of p65 by confocal laser microscopy

Control and GGT1-knockdown BC1 cells were seeded on µ-Slide (Ibidi) and cultured overnight at 37 °C and 5% CO_2_. The next day, after 2 h of cell starvation, the cells were stimulated with 50 ng/mL TNF-α for 0, 15, and 30 min. The cells were then fixed and permeabilized with a Cytofix/Cytoperm kit (BD Biosciences standard protocol) and blocked with 2% BSA for 1 h. The cells were then incubated with anti-p65 antibody as primary antibody for 1 h at room temperature, washed to remove excess primary antibody, and then incubated with anti-rabbit Alexa Fluor 488-conjugated antibody (Life technologies) as the secondary antibody and Hoechst 33,342 nuclear stain (Life Technologies) for 30 min at room temperature. After washing, observations were made on the localization of intracellular proteins using a confocal laser microscope (AxioCam MRm, Carl Zeiss).

### Antibodies

The following antibodies were used: anti-GGT1 (ab88864 for western blotting, Abcam), anti-GTF2I-C-terminal (ab135619 for immunohistochemistry, Abcam), anti-p65 (C-20, Santa Cruz), anti-phospho-p65 (Ser536 93H1, Cell Signaling Technology), anti-IκBα (Cell Signaling Technology), anti-phospho-IκBα (Ser32/36 5A5, Cell Signaling Technology), anti-STAT3 (Cell Signaling Technology), anti-p-STAT3 (Cell Signaling Technology), anti-FLAG M2 (Sigma-Aldrich), anti-c-Myc (Sigma-Aldrich), Alexa Fluor 488 goat anti-rabbit IgG (H + L) (Invitrogen), Alexa Fluor 546 goat anti-mouse IgG (H + L) (Invitrogen) and Hoechst 33,342 trihydrochloride trihydrate (Life Technologies).

### Western blotting and immunoprecipitation

Control (MOCK), GGT1-knockdown cells and cells transfected with GGT1 or GGT1 mutants were lysed with lysis buffer [50 mM Tris–HCl (pH 7.4), 150 mM NaCl, 1% Nonidet p-40 and 3 mM EDTA] containing 1/100 volume of protease inhibitor and phosphatase inhibitor cocktails (Sigma-Aldrich). Sodium dodecyl sulfate (SDS)–PAGE was subsequently performed, and the proteins were transferred to a polyvinylidene fluoride membrane (Merck Millipore). Immunoblotting was performed according to the manufacturer’s protocols.

### Dermatitis induction model and evaluation of inflammation score

Wild-type mice (C57BL/6) were purchased from Japan SLC Co. Mice were kept in a room temperature-controlled, specific-pathogen-free room at the Institute for Genetic Medicine, Hokkaido University. The animal experiments used in this study were approved by the Institutional Animal Care and Use Committees of Hokkaido University (#23-0129) and the ARRIVE guidelines. Mice 6–8 weeks old at the start of the experiment were used. Inflammation scores in the dermatitis model were determined according to a previously published evaluation method^36^. 9.4 µL (20 µM) siRNA (specific for GGT1 (Dharmacon, L-045756-01-0005, ON-TARGETplus Mouse Ggt1 (14,598) siRNA), NF-κB siRNA specific for p65 (Dharmacon, L-040776-00-0005, ON-TARGETplus Mouse Rela (19,697) siRNA), or non-target siRNA (Dharmacon, D-001810-10-05, ON-TARGETplus Non-target TARGETplus Non-targeting Pool) with 5.6 µL cream (Johnson & Johnson) and 5.0 µL saline was applied to the front and back of the ears of C57BL/6 mice on day 0, and then Toll-like receptor 7 (TLR7) (imiquimod; Mochida Pharmaceuticals, Japan) on days 1, 2, and 3. Dermatitis was assessed by measuring the thickness of the ear skin with a micrometer and the amount of change from the original thickness. The measurements were taken up to day 8 and double-checked by an author and another researcher. Humane endpoints requiring euthanasia prior to the experimental time point included very marked erythema, scaling, or thickening, or inflammation of the ear area due to scratching behavior, which resulted in abnormal sores and bleeding, compared to other individuals. Mice were euthanized by cervical dislocation after anesthesia by inhalation of excess 4% isoflurane after scoring was completed.

### Statistical analysis

Experimental data are presented as means ± standard error of means (Mean ± SEM); Student's t-test (two-tailed) or chi-square test was used to test for significant differences between two groups. For propensity score matching of the patient background, EZR was used. A *p*-value < 0.05 was considered significant.

### Supplementary Information

Supplementary Information.

## Data Availability

Single-cell RNAseq analysis data shown in supplementary Fig. 2 are publicly available at THE HUMAN PROTEIN ATLAS (https://www.proteinatlas.org/). Heatmap analysis of pancreatitis-associated genes is available for GGT1 (https://www.proteinatlas.org/ENSG00000100031-GGT1/single+cell+type/pancreas), PRSS1 (https://www.proteinatlas.org/ENSG00000204983-PRSS1/single+cell+type/pancreas), CASR (https://www.proteinatlas.org/ENSG00000036828-CASR/single+cell+type/pancreas), CTRB1 (https://www.proteinatlas.org/ENSG00000168925-CTRB1/single+cell+type/pancreas), ABO (https://www.proteinatlas.org/ENSG00000175164-ABO/single+cell+type/pancreas), SPINK1 (https://www.proteinatlas.org/ENSG00000164266-SPINK1/single+cell+type/pancreas), and CTRC (https://www.proteinatlas.org/ENSG00000162438-CTRC/single+cell+type/pancreas).
