# Supplementary material for: GGT1 is a SNP eQTL gene involved in STAT3 activation and associated with the development of Post-ERCP pancreatitis
Source: Sci Rep. 2024 May 28;14:12224. doi: 10.1038/s41598-024-60312-2 (PMC11133343; doi:10.1038/s41598-024-60312-2)
Supplement: Supplementary file 1 — Supplementary Information. [file 41598_2024_60312_MOESM1_ESM.pdf]

Supplementary Appendix

This appendix has been provided by the authors to give readers additional information about their work.

GGT1 is a SNP eQTL gene and involved in the development of Post-ERCP Pancreatitis via IL-6-STAT3 activation

Ryutaro Furukawa[1,2#], Masaki Kuwatani[2#], Jing-Jing Jiang[1#], Yuki Tanaka[1,3#], Rie Hasebe[1,4], Kaoru Murakami[1], Kumiko Tanaka[1], Noriyuki Hirata[1], Izuru Ohki[1,3], Ikuko Takahashi[1], Takeshi Yamasaki[1,4], Yuta Shinohara[1], Shunichiro Nozawa[1,2], Shintaro Hojyo[1,3,6,] Shimpei I Kubota[1,3], Shigeru Hashimoto[1,6], Satoshi Hirano[5], Naoya Sakamoto[2], Masaaki Murakami[1,3,4,5,6*]

[1]Division of Molecular Psychoneuroimmunology, Institute for Genetic Medicine and Graduate School of Medicine, Hokkaido University, Sapporo, Japan.

[2]Department of Gastroenterology and Hepatology, Hokkaido University Faculty of Medicine and Graduate School of Medicine, Sapporo, Japan.

[3]Group of Quantum Immunology, Institute for Quantum Life Science, National Institute for Quantum and Radiological Science and Technology (QST), Chiba, Japan

[4]Division of Molecular Neuroimmunology, National Institute for Physiological Sciences, Okazaki, Japan

[5]Department of Gastroenterological Surgery II, Hokkaido University Faculty of Medicine, Sapporo, Japan

[6]Institute for Vaccine Research and Development（HU-IVReD）, Hokkaido University, Sapporo 001-0020, Japan

# equal contribution

[*]Correspondence

Masaaki Murakami

Kita-15, Nishi-7, Kita-Ku, Sapporo Hokkaido 060-0815, Japan

Phone: 011-706-5121

E-mail: murakami@igm.hokudai.ac.jp

TABLE OF CONTENTS

Table S1. Primers for qPCR analysis…………………………………………………...4
Table S2. Primers for SNP genotyping……………………………………………………5
Supplementary figure 1. Five pancreatitis-related genes are positive-regulators for the IL-6 amplifier……………………………………………………………………………6-7
Supplementary figure 2. Expression levels of several pancreatitis-related genes in pancreatic cell populations……………………………………………………………………8
Supplementary figure legends……………………………………………………………...9

Table S1: Primers for qPCR analysis

Gene	Sequence (5' to 3')	
Human GAPDH	Forward	GAGTCAACGGATTTGGTCGT
	Reverse	CGCTCCTGGAAGATGGTG
Human IL-6	Forward	GGTACATCCTCGACGGCATCT
	Reverse	GTGCCTCTTTGCTGCTTTCAC
Human GGT1	Forward	AAGCAGTGCTCGAAGATTGG
	Reverse	ACACACAACAGGGCTGCAAT
Mouse HPRT	Forward	GATTAGCGATGATGAACCAGGTT
	Reverse	CCTCCCATCTCCTTCATGACA
Mouse IL-6	Forward	GAGGATACCACTCCCAACAGACC
	Reverse	AAGTGCATCATCGTTGTTCATACA

Table S2: Primers for SNP genotyping

Gene	SNP	Sequence (5' to 3')	
ABO	rs8176693	Forward	TCACTTCTCCCCAAACCAGG
		Reverse	AAGGAGGTCAATGGGAAGCC
PRSS1-2	rs2855983	Forward	TTCATATTCACAGGAACAAGCCTTA
		Reverse	ATCACACAGCTGGGCTTTCTTAG
PRSS1-2	rs10273639	Forward	GGAATGCTGAGTCTCCCTTCT
		Reverse	GAGACCGGCTGTTTCCTGCA
CASR	rs1042636	Reverse	CAAGAGCAACAGCGAAGACC
		Reverse	TCCTTGCAGACCTGTTTCCT
CTRB1-2	rs8055167	Forward	GTTTCAGCTCATTCACCTGCA
	and rs8048956	Reverse	CCTTCTGGGGTCATATTCGC
GGT1	rs8135987	Forward	CCTTTGGAGTCTTCTGCAACATA
		Reverse	TTTCATCTGCCATCTTTGTTTTTC
GGT1	rs2236626	Forward	AACAGGCTCTGAGAGGACCA
		Reverse	GCAATCTCTGGGAGAATCCA
GGT1	rs4820599	Forward	CCACCCAGCTCAAGGTGTAT
		Reverse	GCAGTGCTTAACCCAAAGGA
GGT1	rs5751901	Forward	CTTGTTGGATGTGTAACCATAGGC
		Reverse	ATAGTGAGACCCCACTGCAAAAAT

Supplementary figure 1

A

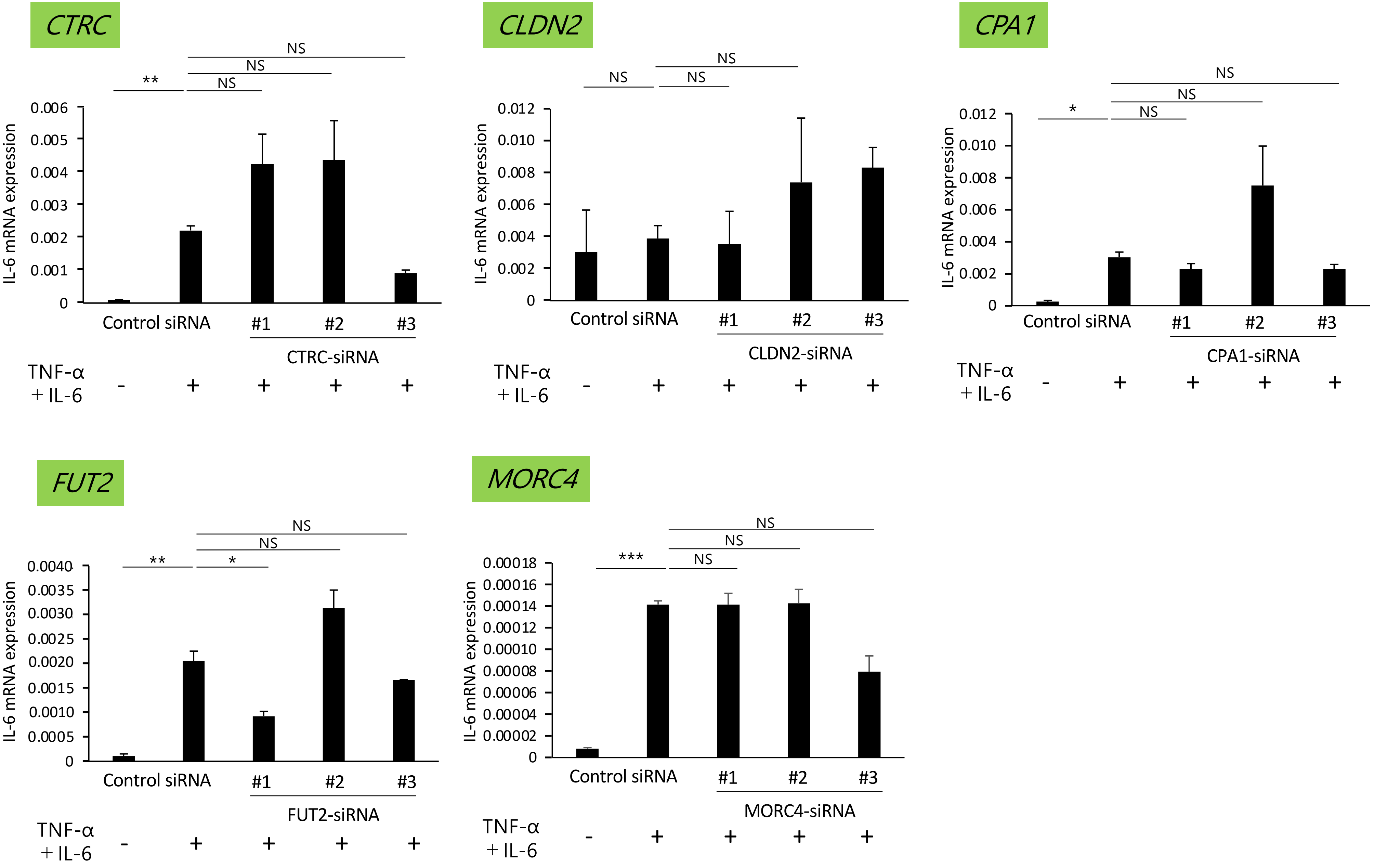

Supplementary figure 1

B

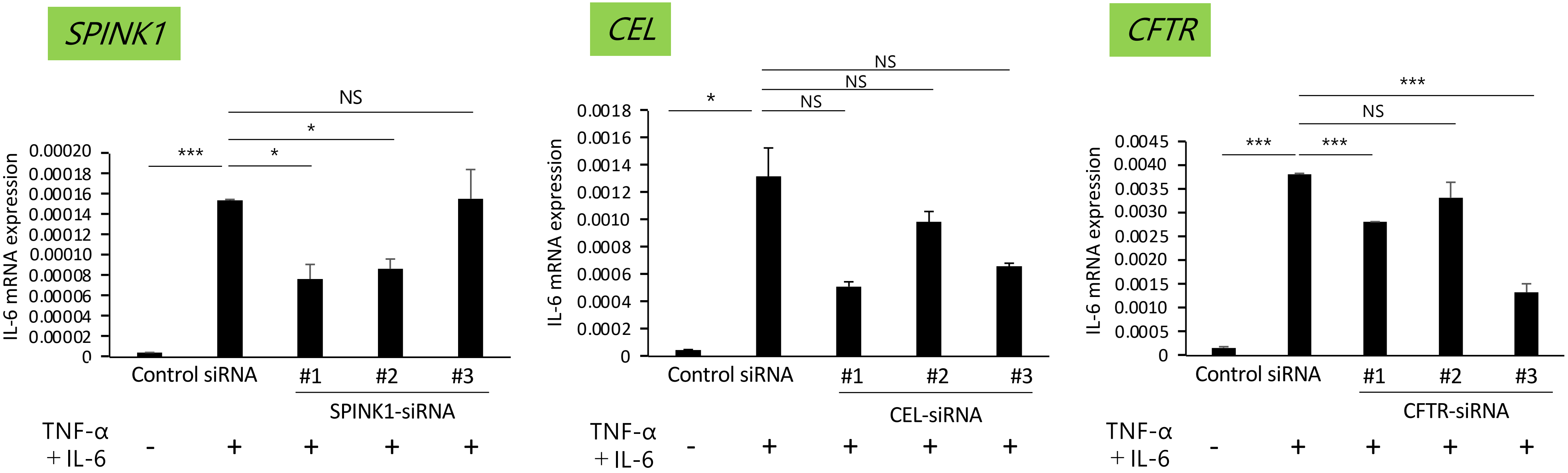

Supplementary figure 2

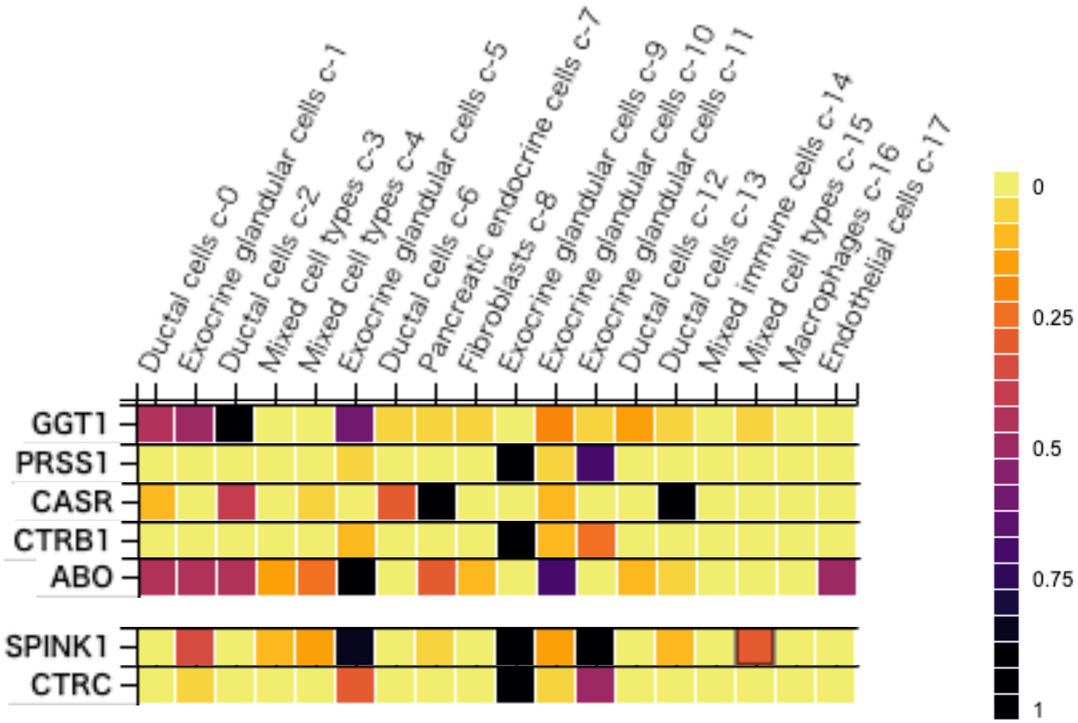

Supplementary figure 1. Five pancreatitis-related genes are positive-regulators for the IL-6 amplifier

(A) H4 cells were transfected with three siRNAs for control, CTRC, CLDN2, CPA1, FUT2, and MORC4, and stimulated with or without TNF-α+IL-6. After the stimulation, qPCR was performed to evaluate IL-6 transcript levels. The silencing of these genes did not effectively suppress IL-6 amplifier. (B) H4 cells were also transfected with siRNAs for control, SPINK1, CEL, and CFTR. The data show the cytokine-induced IL-6 transcript levels. The silencing of these genes partially suppressed IL-6 amplifier.

* $p<0.05$, ** $p<0.01$, *** $p<0.005$; NS, not significant (Student's t-test).

Supplementary figure 2. Expression levels of several pancreatitis-related genes in pancreatic cell populations

Heatmap analysis of pancreatitis-associated genes, whose proteins act as positive regulators of IL-6 amplifier (GGT1, PRSS1, CASR, CTRB1, ABO, SPINK1, and CTRC; **Figure 1A** and **Supplementary figure 1)** by single-cell RNAseq analysis data from THE HUMAN PROTEIN ATLAS (https://www.proteinatlas.org/) are shown.
